# Reparative resynchronization in ischemic heart failure: an emerging strategy

**DOI:** 10.1517/14712598.2014.922536

**Published:** 2014-05-19

**Authors:** Satsuki Yamada, Andre Terzic

**Affiliations:** ^a^ Center for Regenerative Medicine and Division of Cardiovascular Diseases, Department of Medicine, Mayo Clinic, Stabile 5, 200 First Street SW, Rochester, MN 55905, USAterzic.andre@mayo.edu

**Keywords:** biologics, cardiac resynchronization therapy, clinical trial, dyssynchrony, heart failure, myocardial infarction, regenerative medicine, stem cells

## Abstract

Cardiac dyssynchrony refers to disparity in cardiac wall motion, a serious consequence of myocardial infarction associated with poor outcome. Infarct-induced scar is refractory to device-based cardiac resynchronization therapy, which relies on viable tissue. Leveraging the prospect of structural and functional regeneration, reparative resynchronization has emerged as a potentially achievable strategy. In proof-of-concept studies, stem-cell therapy eliminates contractile deficit originating from infarcted regions and secures long-term synchronization with tissue repair. Limited clinical experience suggests benefit of cell interventions in acute and chronic ischemic heart disease as adjuvant to standard of care. A regenerative resynchronization option for dyssynchronous heart failure thus merits validation.

Cardiac dyssynchrony provoked by disparity in contractile timing is a recognized mechanism of poor outcome in patients with heart failure. Cardiac pump function relies on coordinated myocardial motion secured by ordered electromechanical activation. Even in initially healthy hearts, nonphysiological pacing triggers dyssynchrony with detrimental molecular alterations underscoring the requisite of a synchronized contractile pattern for sustained cardiac well-being. Disruption in synchronous motion impacts heart health, compromising vital parameters ranging from ejection volume and diastolic filling to valve function, wall stress and neuro-hormonal activity. Ultimately, cardiac dyssynchrony precipitates structural remodeling and worsens pump failure [1].

Cardiac resynchronization therapy (CRT) through biventricular pacing has offered a major advance in the management of dyssynchronous heart failure [2]. Growing clinical experience demonstrates that device-based CRT produces favorable effects on contractility, reverse remodeling, exercise capacity and overall survivorship. By electrically activating cardiac chambers in an attempt to correct contractile timing, CRT is particularly effective in cardiac dyssynchrony with ventricular conduction delay improving on the efficiency of the contraction–relaxation cycle and supporting hemodynamic performance [3]. Despite documented benefit, current practices that rely on pacing devices are associated with a substantial share of nonresponders among treated individuals [4].

Variance in the magnitude of the response to CRT is not fully understood but is likely due to multiple factors, including idiosyncrasy in the disease substrate. In particular, refractory heart failure is a common outcome of massive myocardial infarction. Prompt revascularization has reduced premature death in the setting of acute myocardial infarction but has produced, in survivors, a high risk for developing chronic heart failure. Cardiac dyssynchrony develops early after successful reperfusion, and is an independent risk factor for heart failure hospitalization and death [5]. Beyond aberrant kinesis due to loss of viable myocardium, discrepancy between infarcted and non-infarcted areas generates an environment conducive to electrical and mechanical discordance contributing, long term, to the heart failure syndrome. Scar formation is notorious in engendering an unfavorable response to device therapy, which critically relies on viable tissue [6,7]. Device-based CRT corrects conduction delays, yet fails to address parenchymal loss that is at the origin of the contractile deficit post-infarction. As a result, the nonviable myocardium remains insufficiently resynchronized by pacing, and dyssynchrony stands uncorrected [8]. The scope of the problem is significant as one-third of qualified candidates who fulfill clinical guidelines for CRT device implantation, or annually roughly a 400-large patient population per million individuals, do not optimally respond [9].

Accordingly, it has been stipulated that restitution of normative impact requires resynchronization in the context of a tissue-reparative solution. With this stringent goal, the notion of ‘reparative resynchronization’ has recently been advanced exploiting the permissive nature of the myocardium for regeneration and the emerging stem-cell toolkit that offers the outlook of genuine structural and functional restoration [10]. Expected to support homeostatic needs, the innate renewal reserve of the human heart is insufficient following myocardial injury. In this context, multiple stem-cell types have been isolated from cardiac and non-cardiac sources or bioengineered to treat ischemic heart disease. By boosting the capacity of the heart to heal, regenerative biotherapies – possibly as adjunct to standard of care – would serve to complement and extend the reach of the existing management armamentarium [11].

In the setting of ischemic heart disease, stem-cell–based therapy has been applied acutely/subacutely after myocardial infarction in an attempt to ensure cardioprotection and delay progression toward ischemic cardiomyopathy, or in florid chronic heart failure as a cardiorestorative strategy to avoid organ decompensation [12]. Beyond the conventional view that transplanted cells directly generate new muscle, recent evidence increasingly highlights an indirect, paracrine mechanism in the repair process that stimulates cross-talk between delivered cells and the diseased myocardium engendering a regenerative response.

To date, cardiac resynchronization post-cell therapy has been tested under several clinical scenarios using autologous stem-cell sources, although the experience remains overall limited (Table 1). In acute myocardial infarction, following drug-eluting stent implantation, intracoronary infusion of peripheral blood-mobilized CD34^+^ cells (90 ± 80 million cells per patient) shows benefit in restoring synchronous left ventricular contraction – exceeding the impact of stent implantation alone [13]. In advanced chronic ischemic heart failure, for patients who were ineligible for coronary intervention, surgery or device therapy, endocardial delivery of bone-marrow–derived mononuclear cells (93 ± 14 million cells per patient) has been linked to reduced dyssynchrony when recovery in left ventricular ejection fraction exceeded 5% [14]. Moreover, combination therapy of bone-marrow–derived mononuclear cells (43 ± 19 million cells per patient) with biventricular pacing was more recently reported in cohorts who met criteria for CRT device implantation [15]. Combined, these apparently complementary therapies improved left ventricular performance in patients with severe heart failure and electrical/mechanical dyssynchrony. Of note, no stem-cell–related adverse effects have been observed in these clinical regimens for cell-based resynchronization that reported absence of either arrhythmogenicity or uncontrolled cell growth [13-15]. Clinical trials have thus established safety and feasibility; however, patient age or comorbidities may compromise the regenerative capacity of utilized stem-cell types, mandating further investigation and optimization [16]. Moreover, side-by-side comparison between stem-cell platforms has not been reported in the setting of cardiac dyssynchrony management, which as such remains largely exploratory.

**Table 1.** **Stem-cell–based cardiac resynchronization studies.**

**Study**	**Cohort Myocardial infarction (MI) Combination therapy Size (n)**	**Cells Type Dose/heart Route of delivery**	**Outcome Follow-up period Echocardiographic readout Efficacy on LV synchrony**
*Clinical*			
Chang *et al*. (2008) [13]	Acute MI	CD34^+^	6 months
	Drug-eluting stent (+)	90 × 10^6^	Tissue Doppler
	n = 40	Intra-coronary	Favorable
van Ramshorst *et al*. (2009) [14]	Chronic MI	BMMC	3 months
	CRT (-)	93 × 10^6^	Speckle tracking
	n = 25	Intra-myocardial	Favorable
Pokushalov *et al*. (2011) [15]	Chronic MI	BMMC	12 months
	CRT (+)	43 × 10^6^	Tissue Doppler
	n = 26	Intra-myocardial	Favorable
*Experimental*			
Bonios *et al*. (2011) [17]	Acute MI model (rat)	CDC	1 month
	Stem-cell monotherapy	2 × 10^6^	Speckle tracking
	n = 14	Intra-myocardial	Favorable
Yamada *et al*. (2013) [10]	Acute MI model (mouse)	iPS	3 months
	Stem-cell monotherapy	200 × 10^3^	Speckle tracking
	n = 56	Intra-myocardial	Favorable

BMMC: Bone marrow-derived mononuclear cells; CDC: Cardiosphere-derived stem cells; CD34^+^: Granulocyte colony-stimulating-factor–mobilized CD34^+^ cells from peripheral blood; CRT: Device-based cardiac resynchronization therapy; iPS: Induced pluripotent stem cells; LV: Left ventricle.

Indeed, clinical studies to date have exploited so-called first-generation stem-cell platforms in combination with standard of care. With the evolution of new technologies, advanced adult stem-cell therapy options or even pluripotent stem cells are increasingly tested in experimental settings (Table 1). Case in point, cardiosphere-derived stem cells, isolated directly from heart tissue, are composed of cell subpopulations with markers of cardiac progenitors, mesenchymal stem cells and endothelial cells that collectively promote cardiac regeneration. Transplantation of cardiosphere-derived stem cells, as a monotherapy, into an infarction model (2 million cells per rat heart) shows improved regional and global contractility with decreased dyssynchrony within infarcted/peri-infarcted regions [17]. Beyond adult stem-cell sources, nuclear reprogramming has provided more recently an unprecedented means to reset cell fate and engineer from somatic tissue, such as a fibroblast, induced pluripotent stem (iPS) cells, which can serve as an unlimited autologous source of new tissue [18]. *In vitro*, iPS cells can differentiate into functional beating syncytia expressing cardiac contractile proteins and ion channel sets responsive to excitation inputs. *In vivo*, iPS cell transplantation achieves, post-injury, multilineage tissue reconstruction [19]. High-fidelity speckle-based imaging has been used to map the transition from the initial focal insult to global dyssynchrony, and assess the responsiveness to therapeutic interventions [20]. Prospective speckle-tracking echocardiography documents the aptitude of targeted iPS cell implantation to rescue contractility and correct discoordination in infarcted regions, a recognized epicenter of dyssynchrony [10]. Initial preclinical experience suggests that dyssynchronous motion characterized by early stretch followed by delayed contraction in the infarcted heart is correctable by iPS cell therapy (200,000 undifferentiated iPS cells per mouse heart; Figure 1). Regional benefit of iPS cell intervention translates into improved left ventricular conduction and contractility, reduced scar and reversal of structural remodeling, protecting against organ decompensation [10]. iPS cells rely on glycolytic metabolism, providing a possible survival advantage within the low-oxygen–containing environment of the ischemic myocardium [21]. *In situ* imaging and *ex vivo* histological validation have implicated iPS cell engraftment and lineage differentiation, pointing to endogenous cell-cycle activation in the diseased heart associated with reduction in fibrotic burden post-infarction [10,19]. Reestablishment of myocardial mechanical properties and correction of coordinated cardiac wall motion offer thereby an integrated readout of myocardial function achieved by tissue repair. Multiple mechanisms of action possibly underlie the benefit of an iPS cell-based intervention, including putative differentiation into cardiomyocytes, vasculature and/or paracrine effects, culminating into induction of an innate regenerative response.

**Figure 1.**
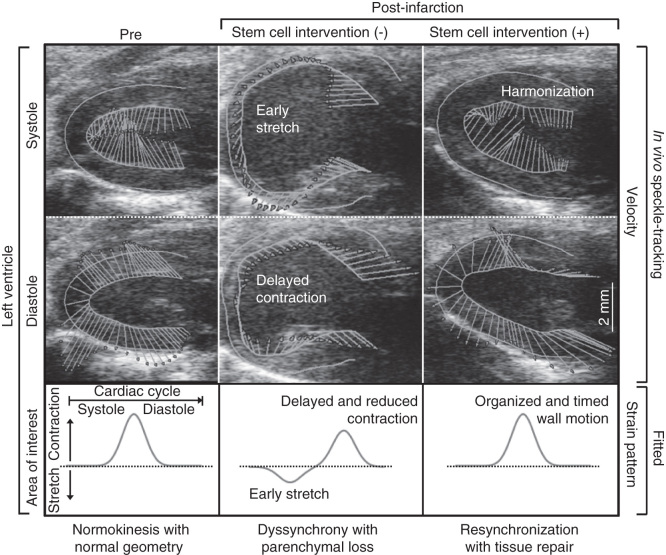
**Stem-cell intervention rescues disparity in ventricular wall motion post-infarction.** Impact of stem-cell biotherapy on cardiac dyssynchrony deconvoluted in a murine infarction model. A total dose of 200,000 undifferentiated induced pluripotent stem (iPS) cells per heart (40,000 cells/site × 5 sites) was delivered by epicardial route into the peri-infarcted anterior wall of the left ventricle within 30 min following coronary ligation. Pre-infarction, all segments of the left ventricle demonstrate harmonious contraction during systole (**left top**) and relaxation during diastole (**left middle**) documented by *in vivo* speckle-tracking echocardiography. At 1 month, infarction precipitated dyssynchronous motion characterized by early stretch followed by delayed contraction (**middle**) with correction afforded by iPS cell therapy (**right**). **Bottom row** depicts fitted strain patterns reflecting normokinesis pre-infarction (**left**), dyssynchrony post-infarction without treatment (**middle**), and resynchronization following cell therapy (**right**). See also Ref. [10].

Translation and adoption of the cell-based cardiac resynchronization principle into practice will require establishment of scalable and standardized stem-cell platforms with robust safety and efficacy profiles, optimized for delivery and tissue implantation in patient populations stratified for maximal benefit. Potential applications of stem-cell–based resynchronization include nonresponders to current management strategies, and prophylactic use as an early intervention for high-risk groups (Figure 2). To this end, establishing validated quality-control procedures through standard operating practices for harvesting, isolation and expansion of cell populations is an essential component in securing desired outcome. Evidence-based and cost-effective procedures will ultimately define an evolving model of regenerative care likely to be implemented to treat selected, well-defined categories of disease and patient populations [22].

**Figure 2.**
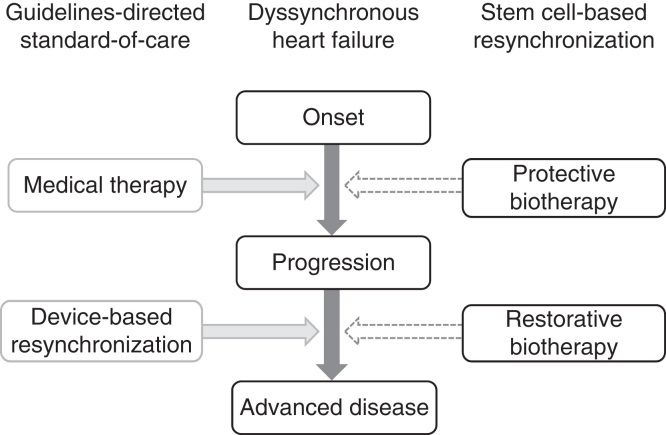
**Stem-cell–based resynchronization complements standard of care.** Dyssynchronous heart failure is a malignant disorder commonly refractory to the existing therapeutic armamentarium that currently combines pharmacotherapy with device-based resynchronization. Responsiveness to pacing devices is impeded by the scar burden post-infarction, mandating approaches capable to promote tissue repair. Potential applications of stem-cell–based reparative resynchronization include cardioprotection in acute/subacute phases of disease to prevent disease progression, and normative restitution to restore structure and function in the setting of chronic dyssynchronous heart failure.

In conclusion, cardiac dyssynchrony is a predictor of poor outcome in the setting of myocardial infarction. However, infarction-induced scar burden impedes an adequate response to device-based CRT. Delivery of stem cells in the acute phase of infarction or with progression of chronic heart failure shows significant potential in reducing the extent of dysfunctional substrates, and prospectively achieving synchronization at the whole organ level. Stem-cell–based resynchronization thus emerges as a promising biotherapeutic strategy equipped to address the primary defects in myocardial pathodynamics that underlie dyssynchronous heart failure post-infarction.

## Expert opinion

Myocardial infarction, a leading cause of heart failure, precipitates dyssynchronous cardiac motion contributing to organ decompensation. CRT, through biventricular pacing, has advanced the management of heart failure. Despite overall benefit, a third of patients does not benefit from a CRT regimen. A culprit underlying unfavorable response to CRT is the infarction-provoked scarburden.

To address refractory dyssynchrony, reparative strategies are increasingly considered. Boosting the repair capacity of the human heart, through stem-cell–based interventions, provides a prospect for functional and structural restoration of the injured myocardium.

Proof-of-concept studies offer initial evidence that transplantation of stem cells may salvage the infarcted myocardium and synchronize failing ventricles. Translation of reparative resynchonization principles into practice will require optimization of the regenerative intervention and stratification of patients most likely to benefit.

The relationship of injury, aberrant wall motion and responsiveness to intervention is yet to be delineated. Establishing best practices is paramount in designing safe and effective protocols tailored to individual patients.

By harnessing the potential of regenerative medicine, stem-cell biotherapy emerges as a potential means to restitute collapsed mechanics in the failing myocardium as a complement to standard of care.

Article highlights.

- Cardiac dyssynchrony, triggered by disruption in coordinated wall motion, contributes to organ failure and poor outcome. Post-infarction, the inhomogeneity across infarcted versus non-infarcted regions generates an environment conducive to development of cardiac dyssynchrony.
- Cardiac resynchronization therapy relies on biventricular pacing, and is integral in managing dyssynchronous heart failure. Yet, the infarction-provoked scar may impede a favorable response to pacing regimens. A nonviable myocardium is inadequately resynchronized by pacing, and dyssynchrony stands uncorrected.
- Restoration of normative impact may require a tissue-reparative strategy. The notion of ‘reparative resynchronization’ was recently formulated highlighting the prospect of stem-cell–based structural and functional repair.
- Nascent experience indicates the promise of regenerative approaches. Preclinically, targeted implantation of stem cells into epicenters of cardiac dyssynchrony translates long term in tissue repair and resynchronization. Clinically, in acute or chronic ischemic heart disease, patients appear to benefit from stem-cell therapy demonstrating on follow-up reduced dyssynchrony.
- Stem-cell–based resynchronization emerges as a biotherapeutic strategy to address primary defects in myocardial pathodynamics underlying heart failure post-infarction, meriting further exploration and validation.

This box summarizes key points contained in the article.

## Declaration of interest

The authors are supported by the American Heart Association, National Institutes of Health, Hitachi, Fondation Leducq, Florida Heart Research Institute, Marriott Heart Disease Research Program and Center for Regenerative Medicine at Mayo Clinic. The authors have no other relevant affiliations or financial involvement with any organization or entity with a financial interest in or financial conflict with the subject matter or materials discussed in the manuscript apart from those disclosed.
